# The Effect of Population Structure on Murine Genome-Wide Association Studies

**DOI:** 10.3389/fgene.2021.745361

**Published:** 2021-09-13

**Authors:** Meiyue Wang, Zhuoqing Fang, Boyoung Yoo, Gill Bejerano, Gary Peltz

**Affiliations:** ^1^Department of Anesthesia, Stanford University School of Medicine, Stanford, CA, United States; ^2^Department of Computer Science, Stanford University School of Engineering, Stanford, CA, United States; ^3^Department of Developmental Biology, Stanford University School of Medicine, Stanford, CA, United States; ^4^Department of Pediatrics, Stanford University School of Medicine, Stanford, CA, United States; ^5^Department of Biomedical Data Science, Stanford University School of Medicine, Stanford, CA, United States

**Keywords:** mouse genetic models, GWAS – genome-wide association study, genetic discovery, population structure, genetic analyses

## Abstract

The ability to use genome-wide association studies (GWAS) for genetic discovery depends upon our ability to distinguish true causative from false positive association signals. Population structure (PS) has been shown to cause false positive signals in GWAS. PS correction is routinely used for analysis of human GWAS results, and it has been assumed that it also should be utilized for murine GWAS using inbred strains. Nevertheless, there are fundamental differences between murine and human GWAS, and the impact of PS on murine GWAS results has not been carefully investigated. To assess the impact of PS on murine GWAS, we examined 8223 datasets that characterized biomedical responses in panels of inbred mouse strains. Rather than treat PS as a confounding variable, we examined it as a response variable. Surprisingly, we found that PS had a minimal impact on datasets measuring responses in ≤20 strains; and had surprisingly little impact on most datasets characterizing 21 – 40 inbred strains. Moreover, we show that true positive association signals arising from haplotype blocks, SNPs or indels, which were experimentally demonstrated to be causative for trait differences, would be rejected if PS correction were applied to them. Our results indicate because of the special conditions created by GWAS (the use of inbred strains, small sample sizes) PS assessment results should be carefully evaluated in conjunction with other criteria, when murine GWAS results are evaluated.

## Introduction

Because of ancestral relatedness among the individuals within an analyzed population, a GWAS will identify a true causative genetic variant along with multiple other false positive associations, some of which arise because of commonly inherited genetic regions within a sub-population. This property, which is referred to as ‘population structure’ (PS) and has been shown to exist in populations ranging from plants (Zhao et al., 2007) to humans (Reich and Goldstein, 2001; Yu et al., 2006), inflates the number of false positive results obtained in a GWAS. Since PS was identified as a significant confounding factor for human GWAS, many methods were developed to distinguish the false positive PS-based associations from the true causative genetic factors for a studied trait. Initially, a *Q+K*(population structure and relative kinship) model (Yu et al., 2006) was used, where *Q* is a matrix that reflects the discrete sub-population for an individual. An improved method for controlling for PS was developed by replacing the *Q* matrix with principle components (PCs) that summarized the genome-wide patterns of relatedness (Zhao et al., 2007). Principal component analysis (PCA) was shown to be useful for inferring PS from genetic data (Price et al., 2006; Yang H. et al., 2011), and the use of PCs for PS capture has been a widely accepted and shown to be an effective method PS correction (Consortium, 2007; Purcell et al., 2007; Yang J. et al., 2011). PCA has two advantages over using the population structure matrix: (i) the finite number of subpopulations do not have to be specified prior to the analysis, which can be an arbitrary process that introduces errors; and (ii) it is far more computationally efficient, which is important when many individuals with many SNPs are evaluated.

Although PS correction methodology has improved and has facilitated genetic discoveries emerging from GWAS of human populations, we do not know whether PS has a significant impact on GWAS analyzing inbred mouse strains. Mouse is the premier model organism for biomedical discovery, and many therapies were initially discovered using mice. Since the inbred laboratory strains are derived from what is estimated to be four ancestral founders that diverged ∼1 million years ago (Guenet and Bonhomme, 2003; Reuveni et al., 2010), PS could certainly impact murine GWAS results and others have advocated that PS correction should be used in murine GWAS (Kang et al., 2008; Sul et al., 2018). However, murine and human GWAS differ in several fundamental ways. A typical human GWAS includes thousands of individuals collected from a natural population. In contrast, while most murine GWAS analyzed less than 30 inbred strains of known ancestry (Beck et al., 2000), the strains are homozygous, they do not inter-breed, and environmental and other variables are tightly controlled. Because of this, the genetic effect sizes examined in murine GWAS are much larger than in human GWAS. Because of these differences, we examined a large database of responses measured in panels of inbred strains to assess the impact of PS on GWAS outcome. For this analysis, we analyzed results obtained using haplotype-based computational genetic mapping (HBCGM), which differs from conventional SNP-based GWAS studies in the type of allelic information analyzed (Zheng et al., 2012). In a conventional murine GWAS, a property of interest is measured in available inbred mouse strains and the phenotypic response pattern is compared with the alleles at individual SNP sites. For HBCGM analysis, the genomic sequence of 49 inbred strains (Supplementary Table 1) was analyzed to produce a database with 25M SNPs (Arslan et al., 2020); and the alleles are organized into blocks with multiple SNPs. Then, genetic factors are computationally predicted by identifying genomic regions (haplotype blocks) where the pattern of within-block genetic variation correlates with the distribution of phenotypic responses among the strains (Liao et al., 2004; Wang and Peltz, 2005; Zheng et al., 2012). HBCGM has successfully identified genetic factors for >22 biomedical traits in mice (Grupe et al., 2001; Rozzo et al., 2001; Liao et al., 2004; Guo et al., 2006, 2007; Liang et al., 2006; Smith et al., 2008; Zaas et al., 2008; Chu et al., 2009; LaCroix-Fralish et al., 2009; Hu et al., 2010a,b; Liu et al., 2010, 2012; Tregoning et al., 2010; Peltz et al., 2011; Sorge et al., 2012; Zheng et al., 2012, 2015; Zhang et al., 2016; Liang et al., 2014; Donaldson et al., 2016; Ren et al., 2020). However, as with other GWAS methods, HBCGM analyses identify many genomic regions with allelic patterns that correlate with a phenotypic response pattern; but only a few contain a causative genetic factor (Zheng et al., 2012). Therefore, we investigated the effect that PS had on murine GWAS results, and the utility of applying a PS association test for eliminating false positives from candidate genes identified by HBCGM. We also examined the potential impact of PS association test on SNP-based GWAS studies.

## Results

**FIGURE 1:**
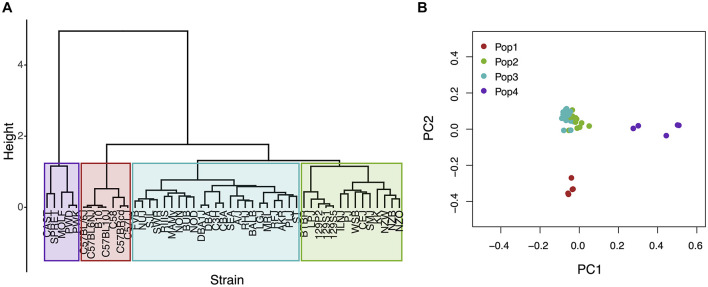
An analysis of population structure among 49 inbred mouse strains, which is based upon whole genome sequence analysis, identifies four sub-populations. **(A)** The relatedness of the 49 inbred strains based upon hierarchical clustering using an identity-by-state similarity matrix; or **(B)** a scatter plot generated by PCA using the first two PCs for each strain are shown. The sub-populations identified by the two methods are completely concordant. Sub-populations 1 and 4 are distinct from the majority of the inbred strains that contained in two closely related sub-populations (2 and 3).

The Mouse Phenome Database (**MPD**)^1^ (Grubb et al., 2014) contains 8223 datasets that characterize basal, age-related, and experimentally induced responses (i.e., ‘phenotypes’) in panels of inbred mouse strains. For each individual MPD dataset, the same response is measured in a panel of inbred strains, and this database has a total of 1.52 M individually measured responses. We previously demonstrated that MPD datasets have utility for genetic discovery; a genetic susceptibility factor for a drug-induced CNS toxicity was identified by HBCGM analysis of one MPD dataset (Zheng et al., 2015). Therefore, we initially examined all MPD datasets that measured a response in 10 or more strains whose genomic sequence was available (2435 datasets). For each of these datasets, candidate haplotype blocks with allelic patterns that correlated with the measured strain response pattern were identified by HBCGM. The average number of correlated blocks (*p*_HBCGM_ < 0.01) for each dataset was 3966, which were selected from among the 6 to 50 million haplotype blocks produced by the algorithm for each dataset. The number of assembled blocks depended upon the number of strains analyzed in a dataset. We then wanted to use a multi-variate association test (MANOVA) to determine whether the haplotypic strain groupings within the correlated blocks were related to PS among the analyzed strains. However, to use PCA for the PS association test, the number of PCs must be specified in advance. Therefore, we first examined the percentage of the variance that was explained when a variable number of principal components (PCs), which ranged from 1 to 33 because ≤33 inbred strains were analyzed in any dataset, were used for the PCA analysis. Because the curves on the Scree plots for most of the evaluated datasets had a bend (i.e., ‘elbow’) that occurred between the 3rd and 5th PC, we used the first four PCs (total genetic variance ranged between 26–59%) as the response variable that was used for the PS association analyses (Supplementary Figure 1). A pairwise identity-by-state (IBS) matrix divided the 49 sequenced inbred strains into four sub-populations (Table 1 and Figure 1), which are based upon their genome wide genetic relatedness. The sub-population grouping, which is based upon the IBS matrix, provides a pre-determined label that is used in the subsequent analyses. Sub-populations 2 and 3 contain most of the inbred strains, and they are closely related. The sub-population 1 strains are derived from a C57BL ancestor; and the five (wild derived) strains in sub-population 4 are genetically distinct from the other groups. The spatial relationship of the 49 strains (plotted using the first two PCs for each strain) is concordant with the hierarchical clustering (Figure 1). A separately performed quantitative analysis (Patterson et al., 2006), which generates Tracy-Widom (TW) statistics and ANOVA values for the groupings, confirms that two PCs captured the PS for these strains (Supplementary Tables 2A,B).

**TABLE 1:** The 49 inbred strains can be divided into the four groups shown in this table based on their pattern of genome-wide allelic sharing.

**Group**	**Number of Strains**	**Strain List**
1	7	C57BL/6J, B10, C57BL10J, C57BL6NJ, C57BRcd, C57LJ, C58
2	14	BTBR, CEJ, KK, NZB, NZW, 129P2, 129S1, 129S5, ILNJ, LPJ, NZO, PJ, SMJ, WSB
3	23	BUB, DBA1J, FVB, NON, NUJ, RFJ, RHJ, RIIIS, SJL, A/J, AKR, BALB, C3H, CBA, DBA, LGJ, MAMy, MRL, NOD, PLJ, SEA, ST, SWR
4	5	CAST, MOLF, PWD, PWK, SPRET

Our global analysis of strain relationships used all available SNPs (25M) to generate the PCs. We also examined the results obtained after LD pruning (PLINK 1.90) of genome-wide SNPs was performed using different window sizes (10 kb, 50 kb) and pairwise correlation coefficients (*r*^2^ = 0.5 or *r*^2^ = 0.75). These analyses utilized 1/10 or 1/5 of the total number of available SNPs. The LD pruned SNPs separated the 49 strains into the same 4 subpopulations, which were found when all SNPs were used (Supplementary Figures 2A–D). Also, even after the removal of the group four wild-derived strains, the PCA plot for the 44 classical inbred strains has the same 3 sub-groups, which were present when all 49 inbred strains were evaluated (Supplementary Figure 2E).

### Most Inbred Strain Panels Have Little or No PS

We then examined PS among the strain panels used in the MPD datasets. The number of inbred strains analyzed in each of the 2435 MPD datasets, which contain data for > 10 evaluable strains, are summarized in Supplementary Table 3. During our analysis, we noted that many different MPD datasets used the same panel of inbred strains, which is because multiple phenotypes were evaluated by the same investigator, and because certain strains are commonly used by different laboratories. Therefore, we could examine PS among the strains used in the majority (55%) of the 2435 MPD datasets by examining the 21 sets of inbred strains that were repeatedly used (Supplementary Table 2C). Our initial analysis of the PS graphs indicated that we should not assess population structure in MPD datasets that analyzed ≤20 strains because: (i) the population substructure was extremely variable, and (ii) the strain groupings within these datasets often contained strains from different global sub-groups (Supplementary Figure 3). To confirm these visual observations, we used the EIGENSOFT/smartpca program (Patterson et al., 2006) to analyze PS in the panels with ≤20 inbred strains, since it provides an unsupervised analysis that ignores the pre-determined of sub-population for each strain. The results indicated that the strain groupings did not have significant PS: all TW test *p*-values were far above 0.05 for the first two PCs (Supplementary Table 2C). Also, the TW *p*-values decreased as the strain number increased, which indicates that it is easier to identify PS when a larger number of strains is evaluated. Overall, only 3 of the 22 strain panels that were repeatedly evaluated in MPD datasets had a TW *p*-value < 0.05 for the first PC; and these 3 panels had over 29 inbred strains and the TW *p*-value for the second PC was not significant (Supplementary Table 2C). These results indicate that most of the strain panels used in the MPD do not have PS that needs to be corrected; and among the few that do, the PS among the strains is not large enough for principal component analysis (PCA) to capture it.

We then examined population sub-structure in the 1750 MPD datasets that examined responses in >20 inbred strains. To illustrate the general properties that emerged from our analyses, we show 960 MPD datasets that repeatedly analyzed responses in the same sets of (*n* = 23-32) inbred strains. The first two PCs for 432 of these datasets did not identify significant PS; there were no clear groupings for the strains; and the TW *p*-values are all > 0.05 (Supplementary Figure 4 and Supplementary Table 2C). In contrast, the PCA plots indicated that PS could be present in 528 other MPD datasets (Supplementary Figure 4) where the group 1 strains (C57BL related) are clearly separated from the other strains. However, in those datasets, the global group 2 and group 3 strains are broadly distributed in the graphs, without an explicit boundary that separates them into distinct sub-groups. It should be noted that 256 of these 528 datasets use two recurring strain panels: 178 datasets use the same 24 strain panel and 78 datasets use the same 25 strain panel (Supplementary Figures 5A,B). Also, the TW *p*-values are > 0.05 for the first two PCs (Supplementary Table 2C) for most of these recurring panels irrespective of whether the strains are separable on the PCA plots. Of importance, even for the datasets that utilize strain panels that appear to have PS, it will only have an effect if the strain grouping for the phenotypic response pattern completely mirrors that the groupings within the sub-populations determined by genome wide analysis of their pattern of allelic sharing.

### PS Impact on Haplotype Blocks

**FIGURE 2:**
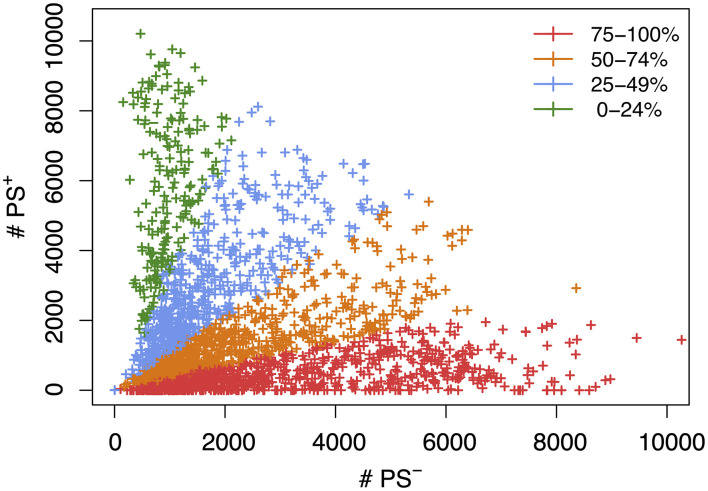
A scatter plot showing the number of candidate haplotype blocks associated with population structure (PS^+^) relative to PS^–^ candidate blocks. After 2435 MPD datasets were analyzed by HBCGM, candidate blocks (*p*_HBCGM_ < 0.01) were analyzed by an association test to determine whether they were related to population structure among the inbred strains that were analyzed. Each datapoint (+) indicates the number of PS^+^ (*y*-axis) and PS^–^ (*x*-axis) blocks identified for one MPD dataset. There are 949 MPD datasets where 75% to 100% of the blocks are PS^–^ (shown in red); the 711 datasets with 51–74% PS^–^ blocks are shown in orange; and the 565 datasets with 25-49% PS^–^ haplotype blocks are shown in blue; and the 210 datasets with 0–24% PS^–^ blocks are shown in green.

**FIGURE 3:**
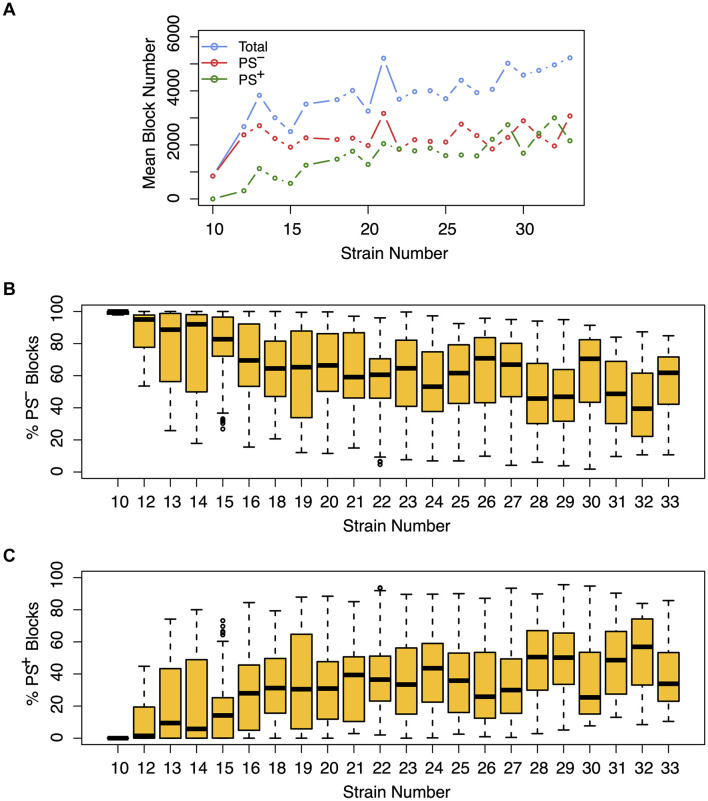
The effect of population structure increases with the number of analyzed strains. Analysis of the total number of candidate haplotype blocks, the number of blocks with population structure (PS^+^), and the number of PS-independent (PS^–^) blocks are shown as a function of the number of analyzed strains. After 2435 MPD datasets were analyzed by HBCGM, the correlated blocks (*p*_HBCGM_ < 0.01) were analyzed by an association test to determine whether population structure had a significant influence on the strain groupings within the blocks. **(A)** The results were then graphed as a function of the number of mouse strains within each dataset (range 10 – 33). A blue circle represents the average of the total number of candidate blocks, and the mean number of PS^–^ (red) and PS^+^ blocks (green) are also shown in this graph. **(B,C)** The percentage of **(B)** PS^–^ and **(C)** PS^+^ blocks were then assessed for each dataset. The box plots indicate the 25th and 75th percentile, and the black bar indicates the median value. While the number of PS^–^ blocks plateaued after 15 strains were analyzed, the number of PS^+^ blocks increased in the datasets that analyzed an increased number of strains.

We next assessed the impact of PS on the haplotype blocks generated by HBCGM analysis. To do this, a PS association test was performed on each correlated haplotype block produced from the analysis of the 2435 MPD datasets with phenotypic data covering >10 strains. A Benjamini-Hochberg adjusted *p*-value for the PS association test for each block was generated using MANOVA. Blocks with a *p*_adj_ < 0.05 have a significant association with population structure (i.e., PS^+^), and could be removed from further consideration, while those with a *p*_adj_ > 0.05 are viewed as viable candidate genes for further evaluation (PS^–^). For 68% of the datasets (1,660 of 2435 analyzed), >50% of the correlated blocks were not associated with population structure (PS^–^); and 39% of the datasets (949 of 2435) had 75 to 100% PS^–^ blocks (Figure 2). Only 32% of the datasets (*n* = 775) had >50% PS^+^ correlated blocks; and most of these (23%, 565 datasets) have between 25 and 49% PS^–^ blocks. Only 9% of the MPD datasets (*n* = 210) have >75% PS^+^ blocks. Overall, our results indicate that for most MPD datasets, the vast majority of the haplotype blocks produced by HBCGM are not affected by PS. We also investigated whether the magnitude of the PS impact is affected by the number of strains analyzed (i.e., the sample size). As the strain number increased, the number of correlated candidate blocks identified by HBCGM analysis increased (Figure 3A). This result is consistent with prior studies indicating that association tests performed on large populations will identify additional genetic variants with a small effect size (Visscher et al., 2017). However, while the percentage of PS^–^ blocks plateaued after 15 strains were analyzed, the percentage of PS^+^ blocks (and thus the total number of PS^+^ blocks) increased as the number of analyzed strains increased (Figures 3B,C). These results indicate that when an increased number of inbred strains are analyzed, the number of correlated haplotype blocks and the percentage of PS^+^ blocks increase. The results are consistent with the sample size effects previously noted in human-case control studies.

### Assessing the False Negative Problem

**FIGURE 4:**
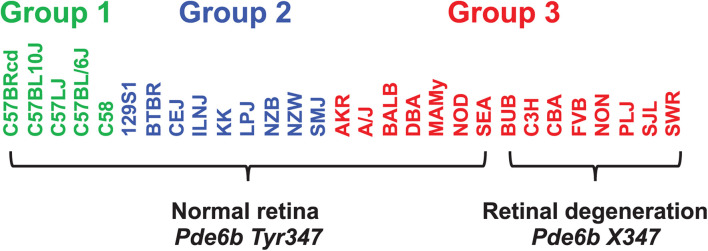
The haplotype block with a causative mutation is associated with population structure. MPD 26721 examined the retinas of 29 inbred strains: 21 strains had normal retinas and 8 strains had retinal degeneration. A haplotype block within *Pde6b* contained the causative SNP (*Tyr347X*) for this type of retinal degeneration. All strains with retinal degeneration had the *Pde6b 347X* allele, while those with normal retinas had the *Tyr347* allele. The haplotype block had PS, because all group 1 and 2 strains [based upon hierarchical clustering of whole genome sequence data from 49 inbred strains (Table 1)] had normal retinas; while all strains with retinal degeneration were group 3 strains. However, several group 3 strains (AKR, A/J, BALB, DBA, MAMy, NOD, SEA) had normal retinas and the *Tyr347* allele. Thus, while the strain groupings within the block have PS based upon their global allele sharing pattern, the allelic pattern within the haplotype block had a stronger association with retinal degeneration.

When considering whether PS correction should be utilized for mouse GWAS, the key question is whether it could lead to rejection of a true causative association signal. Therefore, we investigated whether PS was present in haplotype blocks within genes whose allelic patterns are known to be causal for a measured phenotypic response pattern (Table 2). The results of PS analyses for three MPD datasets raised concerns. (i) HBCGM analysis of two datasets (MPD 9904 and 9907), which measured high density lipoprotein (HDL) cholesterol levels, correctly identified haplotype blocks within *Apoa2* as highly correlated with inter-strain differences in HDL levels. *Apoa2* encodes the second most abundant protein within HDL particles, and it is known to be involved in lipoprotein metabolism. *Apoa2* alleles were previously associated with differences in plasma HDL cholesterol levels in mice (Doolittle et al., 1990); and HDL levels were 70% decreased in *Apoa2* knockout mice (Weng et al., 1999). However, a PS association test indicated that 3 of the 4 correlated haplotype blocks in *Apoa2* are PS^+^ blocks PS (GRM)adj *p*-val < 0.05). (ii) Another MPD dataset (MPD 26721) examined the retinas of 29 inbred strains: 21 strains had normal retinas, and 8 strains had retinal degeneration. HBCGM analysis identified a haplotype block within *phosphodiesterase 6b* (*Pde6b*) that completely correlated with the pattern of retinal degeneration in both male and female mice (*p*_HBCGM_ = 0). Retinal degeneration in inbred strains has been shown to be caused by a stop codon allele (*Tyr347X*) within *Pde6b* (Pittler et al., 1993). However, the strain groupings within the *Pde6b* block were correlated with PS; the PS association test *p*-values for this block was 0.02 (*p*_adj_ = 0.049) (Table 2). The blocks had PS because all 8 strains with retinal degeneration were from population group 3, and all population group 1 and 2 strains had normal retinas. However, several group 3 strains had normal retinas and *Pde6b Try347* alleles (Figure 4). These examples demonstrate that some true positive genetic associations could have been falsely rejected based upon their association with PS (if the usual FDR control rate *q* = 0.05 was applied). We also examined these datasets using the PCs that were derived from an identity-by-state (IBS) matrix that was used to represent the PS (Table 2). The PS association test *p*-values using PCs derived from the IBS matrix are nearly the same as the those obtained using PCs derived from genetic relationship matrix. This concordance indicates that the PCA using different types of marker-derived matrices stably capture the PS for the inbred strains.

**TABLE 2:** The results of PS analysis performed on haplotype blocks within known causative genes for 3 MPD datasets (each with data from both sexes) are shown.

**MPD Dataset**	**Strain #**	**Gene**	**Block Position**	**HBCGM *p*-val**	**PS (GRM) *p*-val**	**PS (GRM) adj *p*-val**	**PS (IBS) *p*-val**	**PS (IBS) adj *p*-val**
26721 F retinal degeneration	29	*Pde6b*	Chr5: 108399551-108400383	0	0.0244	0.0491	0.0274	0.0514
26721 M retinal degeneration	29	*Pde6b*	Chr5: 108399551-108400383	0	0.0244	0.0491	0.0274	0.0514
9904 F HDL cholesterol baseline	30	*Apoa2*	Chr1: 171225795-171225890	5.5e-6	0.0005	0.0010	9.3e-4	0.0026
9904 M HDL cholesterol baseline	31	*Apoa2*	Chr1: 171225644-171225697	3.14e-5	0.1537	0.2448	0.1497	0.2226
9907 F HDL cholesterol after 17 weeks on diet	30	*Apoa2*	Chr1: 171227457-171227593	0.0066	0.0039	0.0106	0.0042	0.0156
9907 M HDL cholesterol after 17 weeks on diet	25	*Apoa2*	Chr1: 171227457-171227593	0.0008	0.0020	0.0044	0.0033	0.0068

*The MPD dataset number, the sex of the mice, a description of the measured response, and the number of strains analyzed in that dataset are shown. The gene symbol for the causative gene, the chromosome and position of the identified haplotype block, and the *p*-value and adjusted *p*-value for the PS association test (using the GRM) for that block are shown. We also calculated an additional *p*-value and adjusted *p*-value for the PS analyses, which were performed using PCs derived from an identity-by-state (IBS) matrix, and these results are shown in the last two columns.*

### PS Impact on Causative SNPs

We also examined whether SNPs or indels, which are known to be causative of biomedically important trait differences among inbred strains, were highly associated with PS. To do this, causative variants were downloaded from a public database that described the published evidence linking the variants to phenotypes (Bult et al., 2019). Surprisingly, we found that four of the 15 evaluable variants in this database were strongly associated with PS among the inbred strains (Table 3). (i) For example, the albino skin and eye hypopigmentation observed in inbred strains were experimentally proven to be determined by a *Cys103Ser* SNP allele within *tyrosinase* (*Tyr*) (Jackson and Bennett, 1990; Yokoyama et al., 1990) (MGI:1855976), but these alleles are very strongly associated with PS among the inbred strains (PS *P*-value = 2 × 10^–4^). (ii) An AGTC sequence insertion (GRCm38/mm10 chr2: 130048178-130048179) in *Transglutaminase 3* (*Tmg3)* (MGI:1856269) produces wavy hair morphology (Brennan et al., 2015), along with 13 other listed traits in mice, but this indel is also very strongly PS associated (PS *P*-value = 4 × 10^–4^) among the inbred strains. (iii) A spontaneous C to A transversion in *Cell Division Cycle 25A (Cdc25A)* increases the activity of a phosphatase (Melkun et al., 2002), which causes abnormal erythropoiesis and increased cell proliferation (MGI:2445422). This *Cdc25A* variant is very strongly associated with PS (PS *P*-value = 3 × 10^–5^). (iv) Deficiencies in phosphatidylcholine metabolism in NZO/HlLtJ mice (and in the related NZB/BlNJ and NZW/LacJ strains) are determined by a C to T mutation within *Phosphatidylcholine transfer protein (Pctp)* (Pan et al., 2006). This causative SNP (MGI:3691424) also had a strong association with PS (PS *P*-value = 8.3 × 10^–10^). If normal GWAS procedures were performed using inbred strains for any of the 44 phenotypes shown in Table 3, the known causative alleles (*Tyr Cys103Ser, Tmg3 Indel, Cdc25A C > A, and Pctp C > T*) would have been eliminated from consideration because of PS correction. The false negatives generated by PS correction would have produced a complete disaster for these GWAS studies, since the causative genetic variation occurred at sites where the alleles were commonly inherited among the inbred strains.

**TABLE 3:** Population structure (PS) analysis was performed on causative SNP alleles for 44 mammalian phenotypes (MP) that were annotated in the Mouse Genome Informatics (MGI) database.

**MGI Mammalian Phenotypes**	**Number of MP Terms**	**Gene**	**Point Mutation Position**	**PS *p*-val**
0002075 abnormal coat/hair pigmentation 0001324 abnormal eye pigmentation 0000371 diluted coat color 0005171 absent coat pigmentation 0005408 hypopigmentation 0011551 variegated eye pigmentation pattern 0011091 prenatal lethality, complete penetrance 0001303 abnormal lens morphology 0001304 cataract 0005643 decreased dopamine level 0003136 yellow coat color 0005077 abnormal melanogenesis 0008480 absent eye pigmentation 0003962 abnormal adrenaline level 0005172 decreased eye pigmentation 0010193 abnormal choroid melanin granule morphology 0001189 absent skin pigmentation 0005075 abnormal melanosome morphology 0000421 mottled coat 0001510 abnormal coat appearance 0011279 decreased ear pigmentation 0000373 belly spot 0003964 abnormal noradrenaline level 0010192 abnormal retinal melanin granule morphology 0004381 abnormal hair follicle melanocyte morphology	25	*Tyr*	Chr7:87493043	1.97 × 10^–4^
0009351 thin hair shaft 0010099 abnormal thoracic cage shape 0003641 small lung 0001274 curly vibrissae 0002113 abnormal skeleton development 0000410 waved hair 0011400 lethality, complete penetrance 0001406 abnormal gait 0001510 abnormal coat appearance 0000162 lordosis 0001177 atelectasis 0003109 short femur 0004703 abnormal vertebral column morphology 0001533 abnormal skeleton physiology	14	Tgm3	Chr2:130048178	3.97 × 10^–4^
0004045 abnormal cell cycle checkpoint function 0005584 abnormal enzyme/coenzyme activity 0000245 abnormal erythropoiesis 0000351 increased cell proliferation	4	Cdc25A	Chr9:109879893	2.96 × 10^–5^
0002118 abnormal lipid homeostasis	1	Pctp	Chr11:89987348	8.3 × 10^–10^

*The MGI MP terms, the number of MP terms associated with the known gene, the chromosome and location (all in GRCm38/mm10 coordinates) of the known causative allele, and the PS P-value are shown.*

## Discussion

While PS correction helps to eliminate false positives in human genetic studies, we found that PS makes a smaller than expected contribution to most murine GWAS studies. Moreover, irrespective of whether a murine GWAS used SNPs or haplotype blocks, our results indicate that PS correction could result in rejection of association signals that were generated by known causative alleles. Of importance, this analysis evaluated the largest available dataset of phenotypic information for inbred mouse strains, and the data was generated by most of the researchers who are studying genetic traits in mice. Why is the utility of PS correction in murine GWAS different from that for human genetic association studies? We identify three factors that could account for this difference. (i) A very limited number of inbred strains are examined in most murine GWAS, which usually analyze < 20 (and rarely > 33 inbred strains). This is orders of magnitude less than the number of subjects in human GWAS, which now examine thousands to hundreds of thousands of subjects. Moreover, the inbred strains were reproductively isolated, while human populations were not placed under this restriction. (ii) The vast majority of murine GWAS studies utilize strains with limited PS. Most (75%) of the inbred strains that are commonly used in murine GWAS are derived from closely related populations, which have limited or no population structure. Among 25M SNPs analyzed, pairwise comparisons revealed that the level of allelic similarity among the classical inbred strains is > 70%. The limited amount of genetic variation among these strains precludes their separation into distinct sub-populations. (iii) A false negative result resulting from exclusion of a true positive due to PS correction has a much greater impact on murine GWAS outcome. Genetic association studies involving large human populations often (but not always) identify many genetic variants, with each having a small effect on the overall trait value. Hence, the loss of a few true positives can have a lesser impact since many other causative loci remain. However, murine GWAS analyze a small number of inbred strains; and the heritability and genetic effect size for identified candidate genes is relatively large (usually > 0.3) because the inbred strain genome is homozygous and because environmental and other confounding factors are minimized. Thus, unlike its small effect on human GWAS results, the elimination of a true positive due to PS correction, which in some cases could be the only (or one of a very few) causative genetic factor, can have a much greater impact on a murine GWAS.

We identified six examples (Pde6b, Apoa2, Tyr, Tgm3, Cdc25A, and Pctp) where PS correction could cause an adverse outcome for murine GWAS. Irrespective of whether haplotype blocks or SNP/Indels were analyzed, PS correction led to rejection of the causative variant due to common inheritance. Other investigators who examined GWAS results for multiple traits in plants have noted that it can be difficult to distinguish between a true and a spurious association due to genetic background, even after correcting for PS (Atwell et al., 2010). However, when GWAS are performed under conditions with true genome wide coverage, allele sharing within a localized genomic region with a true causative factor should be greater than one based upon genome wide allelic correlations. Hence, examining the ratio of the p-values obtained from GWAS and PS association tests could provide a more informative way to eliminate spurious positives while retaining the true positive associations. In one of our studied cases (retinal degeneration and Pde6b), the causative haplotype block was much more strongly associated with the phenotypic response pattern (genetic association p-value = 0) than with population sub-structure (PS p-value = 0.024), but in another case (HDL levels and Apoa2), the p-values for the causative haplotype block were of a similar magnitude. However, published information indicated that the gene candidate (Apoa2) was very strongly associated with the HDL phenotype. As was previously observed in plants (Atwell et al., 2010), and now in mice, there are situations where a shared strain background can be responsible for trait differences. In these situations, the strength of the functional evidence that a candidate gene could be responsible for a trait difference could override PS considerations. We have previously shown that true positive candidates can be identified using orthogonal criteria for analyzing HBCGM output, which include the use of gene expression or metabolomic data (Liu et al., 2010), curated biologic information (Zhang et al., 2011), or the genomic regions delimited by prior QTL analyses (Smith et al., 2008; LaCroix-Fralish et al., 2009). Similar to our approach to mouse GWAS, investigators have recently used transcriptome wide association data (Hammerschlag et al., 2019; Wainberg et al., 2019), information about plant evolutionary type (Liu et al., 2020), or various types of functional information to evaluate human (De Leeuw et al., 2016; Watanabe et al., 2019) or plant (Atwell et al., 2010) GWAS results. In summary, PS assessment may be one factor that should be used along with multiple other factors to assess a candidate gene, which include assessment of the relative strength of the GWAS and PS association results, tissue-specific gene expression criteria, and gene-phenotype relationship based upon information contained within the published literature.

Genetic association studies typically use two different methods to analyze PS (Greenbaum et al., 2016): (i) phylogenetic methods based on pedigree and evolutionary history (Pickrell and Pritchard, 2012; Liu et al., 2020) or (ii) clustering of the individuals into subpopulations based on their genetic relatedness, which can be further divided into model- and distance-based approaches (Greenbaum et al., 2016). The model-based approaches assume that individuals are drawn from a predefined number of subpopulations (Pritchard et al., 2000), which are in Hardy-Weinberg equilibrium. While distance-based approaches (such as PCA) focus on the genetic differences or similarity between individuals, they do not require prior assumptions. Over past decade, distance-based methods became much more widely utilized for capturing PS and for assessing cryptic relatedness (Wen et al., 2018, 2019; Wang and Xu, 2019; Wang et al., 2020); and the kinship matrix was used to derive PCs, which can be obtained by evaluation of identity by descent (IBD) or calculation of relatedness based on marker data (Astle and Balding, 2009). The inbred mouse strains (Swiss mice, Castle’s mice, C57 related strains, etc.) were isolated by different laboratories beginning over ∼100 years ago, and those in each category underwent an unclear breeding process that extended over a long period of time. Because of these unknowns, significant uncertainties are introduced when transforming the information about inbred strain phylogeny obtained from pedigree charts into the parameters that are required for evolutionary history-based PS associations. Hence, we cannot use evolutionary history-based methods for assessing PS among the inbred strains.

Various recombinant inbred (RI) strain panels have been used for genetic mapping studies: the Hybrid Mouse Diversity Panel (30 founder strains) (Tewhey et al., 2011; Ghazalpour et al., 2012); the Diversity Outbred (Chick et al., 2016) and Collaborative Cross (Chesler et al., 2008) panels (8 strains); and the BXD RI panel (Belknap and Crabbe, 1992) (2 strains). Since all founder strains for these RI panels are a subset of the strains evaluated here, our cautions about the utilization of PS correction methods may be relevant to studies performed using these RI panels. While these RI panels have proven useful for genetic mapping, GWAS that cover a wider set of inbred strains will always be needed for 21st century genetic discovery. We do not know which strains will have the outlier (disease-related) phenotypes - and they may not be among the founder strains for existing RI panels - that are needed to uncover the genetic basis for biomedical traits that will be of interest over the next 25 years. As one example, Type 2 Diabetes Mellitus (T2DM), and its principal risk factor (obesity) have become a major 21st century public health problem (Centers for Disease Control and Prevention, 2020). The TallyHo strain is not among the founder strains used for the any of the current RI panels, but it provides a valuable murine model for T2DM and obesity because its spontaneously develops hyperlipidemia, hyperglycemia, insulin resistance, and glucose intolerance (Kim et al., 2001; Kim and Saxton, 2012). Undoubtedly, other inbred strains will be identified to have phenotypes reflecting 21st Century diseases.

## Methods

### Selection of Mouse Phenome Database Datasets

Mouse Phenome Database datasets (n = 8223) were downloaded on March 24, 2020. We analyzed MPD datasets where the mean phenotypic measurement of each strain was obtained from >5 mice of each strain. An ANOVA test was also performed to determine if the inter-strain variance was significantly greater than intra-strain variances; and a p-value < 1 × 10^–10^ was used as the cutoff for dataset selection. Datasets with categorical measurements were excluded from bulk analysis of MPD datasets.

### Haplotype Block Construction and Genetic Mapping in Mice

The genomic sequences of 49 inbred mouse strains were analyzed as previously described (Zheng et al., 2015). Only SNPs meeting the following criteria were used for haplotype block construction: (i) polymorphic among the strains with input trait data; and (ii) there were at least 8 strains with unambiguous allele calls, which is an important criterion because it ensures that there is sufficient genetic diversity in the analyzed cohort for analysis by HBCGM. In brief, SNPs were dynamically organized into haplotype blocks, which only used alleles for the strains contained within the dataset, according to the “maximal” block construction method (Peltz et al., 2011). In brief, this method produces haplotype blocks with a minimum of 4 SNPs; and each block is only allowed to a predetermined number of haplotypes, which ranges from 2 to 5. Since the “maximal” method enables blocks to overlap, blocks are assembled that cover all possible allelic combinations within a specific genomic region. If a smaller block was nested inside of a larger block and it contained the same haplotypes, it was removed and the larger block was used to cover that region (Peltz et al., 2011). This ensures that additional SNPs are only included within a block if additional haplotypes are added to the block. HBCGM was then performed as originally described (Liao et al., 2004) using modifications described in Peltz et al. (2011). Haplotype blocks with 2, 3, 4 or 5 haplotypes were then dynamically produced and the correlation between the input phenotypic data and the haplotype pattern within each identified block was evaluated as described as described (Peltz et al., 2011). The genes are then sorted based upon the ANOVA p-value (in increasing order) for numeric data or by the F statistic (in decreasing order) for categorical data. A cut-off of *p* = 0.01 was used to select haplotype blocks with a correlated allelic pattern. If a gene had multiple correlated blocks, the haplotype block with the smallest p-value was used. Additional details about the HBCGM method are described elsewhere (Wang and Peltz, 2005; Zheng et al., 2015).

The genetic effect size (η^2^) is calculated:


$$\eta^{2}=\frac{\sigma_{B}^{2}}{\sigma_{\text{T}}^{2}}=\frac{S\,S\,B}{S\,S\,T}$$

where SSB is the between-group sum-of-squares of the ANOVA model given as and SST is the total sum-of-squares. η^2^ is the genetic effect of the groups defined by haplotypes on the trait value and the total variance ($\sigma_{T}^{2}$) consists of within-group variance and between-group variance given as:


$$\sigma_{\text{T}}^{2}=\sigma_{\text{B}}^{2}+\sigma_{\text{W}}^{2}$$

For a sample size of n with *k* groups, with equal group sizes the *F* statistics of samples with effect size η^2^ follows a noncentral *F* distribution as *F*(k – 1, n – k, λ) with the non-centrality parameter:


$$\lambda =n\,\sigma_{\text{B}}^{2}\,/\,\sigma_{\text{W}}^{2}=n\,\sigma_{\text{B}}^{2}\,/\left(\sigma_{\text{T}}^{2}-\sigma_{\text{B}}^{2}\right)=n\,\eta^{2}\,/\left(1-\eta^{2}\right)$$

Therefore, the significance level α for power of one-way ANOVA test is given as:


$$\text{Power}({\text{α,η}}^{2},\text{n,k})=\text{Prob}(F(k-1,n-k,\lambda ))<F_{\text{crit}})$$

where *F*_crit_ = *F*_(1–α, k–1, n–_*_k_*_)_ is the (1-α) quantile of the *F* distribution with *k* – 1 and n – *k* degrees of freedom.

### Population Structure Association Test

We use principal component analysis (PCA) to determine whether a haplotypic strain grouping was associated with PS. Principal components (PC) have been used to assess population stratification; it is a major component of the linear mixed model (LMM) that is used to control PS-induced spurious associations in GWAS results. In the LMM, PS is treated as a covariate that influences the phenotypic values in addition to the effect of the genetic markers. However, we treat PS as a dependent variable, which is determined by a comprehensive analysis of genome-wide allelic similarity. For this analysis, the PS of the inbred strains (*y*) is determined by the equation


y=μ+Xβ+e

where *y* is an *n*×*p* matrix that is derived from a PCA of sample size of *n* with *p* principal components; μ is an *n*×*p* matrix that contains the grand mean for each of the *p* variables; *X* is an *n*×1 vector of haplotype indicators for *n* strains; β is a 1×*p* vector that contains effects of the haplotype, and *e* is an *n*×*p* matrix of the residual error. *p* is a hyperparameter to determine the number of PCs used in analysis, where it guarantees each PC can explain certain amount (say > 5%) of the variance of the original genetic relationship. Alternatively, *p* can be arbitrarily selected based upon analysis on a Scree plot (to find the “elbow”), which ranks PCs based on the percentage of variance explained by each PC. If the elbow is observed at *p*-th PC; most of the true signals are captured in the first *p* PCs. By using PC to represent population structure, pre-determination of the number of sub-populations is not required. A multivariate analysis of variance (MANOVA) could be then used to assess the association between strain groupings within a haplotype block and PS, since the strain grouping within a block becomes a single variable that affects the first *p* PCs. In this study, the PCs are the eigenvectors of the genetic relationship matrix (GRM) for the inbred mouse strains, which is also known as the variance-covariance standardized relationship matrix.

### Population Structure Analysis on Single Point Mutations

The MGI PostgreSQL database (Bult et al., 2019) was queried for sequence variants linked with Mammalian Phenotype (MP) terms. There were 463 spontaneously occurring sequence variants (i.e., not mutagen induced) that were annotated with 2,878 MP terms. However, after excluding 51 allelic variants that appeared in C57BL/6 because it is the reference strain; only 30 of these SNP alleles and Indels, which were associated with 429 MP terms, were present in our 48 other strains. We also had to remove rare variants present in < 3 strains (i.e., had minor allele frequency < 0.05) because they could not be used for PS analysis. The remaining 15 evaluable variants, which were associated with 155 MGI MP terms, were used for the PS association analysis. The PS association test was performed on these alleles as described above; except the *X* and β term in the linear equation were replaced with the strain allele indicator and the effect of that allele, respectively.

### Generation of Genetic Relationship and Identity-By-State Similarity Matrices

The genetic relationship matrix (GRM) for inbred mouse strains was generated using genome-wide SNP alleles and GCTA software (Yang J. et al., 2011). The GRM is also known as the variance-covariance standardized relationship matrix, and the eigenvectors of this matrix were used as PC. The GRM eigenvalues for the inbred strains of each PC were used to estimate the amount of GRM variance that PC explains. To assess whether a PC effectively captures the sub-structure of the GRM, the Tracy-Widom (TW) statistic and corresponding *p*-values were calculated using EIGENSOFT/smartpca program (Patterson et al., 2006). This program provides an unsupervised analysis, which ignores the pre-determined global sub-populations identified for each strain. Since we analyze 49 inbred strains whose genomes are homozygous, SNPs were not filtered based upon a minor allele frequency threshold. To further verify that the PCs effectively represent the PS among the strains, we clustered individual strains using a pairwise identity-by-state (IBS) similarity matrix, which was also derived using whole genome SNP data. The IBS similarity matrix is a square, symmetric matrix that reflects the IBS distance between all pairs of inbred mouse strains. PLINK 1.90 (Purcell et al., 2007) was used to calculate the IBS similarity matrix, and it contains values that range from 0 to 1. The hierarchical clustering of 49 strains was determined using the hcut() function within the factoextra/R package^2^. The sub-population of an inbred strain is based upon its genetic relatedness relative to the other 49 strains. This clustering determines the sub-population for a strain used in subsequent analyses (i.e., their pre-determined label). Then, an ANOVA test is used to evaluate the overall genetic differentiation between any two pre-determined sub-populations along the PCs (i.e., it is a supervised analysis). Hence, the basis for the 4 sub-populations identified using the IBS similarity matrix for the 49 inbred strains can be assessed using the ANOVA test, where the resulting ANOVA *p*-value is compared with 0.05.

### Multiple Test Correction for the PS Association Test

Since the population structure association test was performed on 2435 datasets, the MANOVA test *p*-value for each block generated by the HBCGM program is adjusted by controlling for the false discovery rate (FDR) at *q* = 0.05 using Benjamini-Hochberg method (Benjamini and Hochberg, 1995). The adjusted *p*-value for i-th block is *p*_adj_ = *p*_i_×*m*/*i*, where *p*_i_ is the MANOVA test *p*-value, *m* is the number of blocks (multiple tests), and *i* is the order of *p*_i_ in a series of *p*-values that satisfies *p*_(1)_≤*p*_(2)_≤⋯≤*p*_(*m*)_. If a block has *p*_adj_≥0.05, it is not considered as having significant PS (i.e., the null hypothesis, which is that the tested block does not have population structure, cannot be rejected).

## Data Availability Statement

The original contributions presented in the study are included in the article/Supplementary Material, further inquiries can be directed to the corresponding author/s.

## Author Contributions

The project was formulated at working meetings of all authors. MW analyzed the data, and BY and GB helped with the analysis. BY and ZF contributed code. GP and MW wrote the manuscript with input from all authors. All authors contributed to the article and approved the submitted version.

## Conflict of Interest

The authors declare that the research was conducted in the absence of any commercial or financial relationships that could be construed as a potential conflict of interest. Reviewers YX, JW, and YC declared a past co-authorship with one of the authors MW to the handling editor.

## Publisher’s Note

All claims expressed in this article are solely those of the authors and do not necessarily represent those of their affiliated organizations, or those of the publisher, the editors and the reviewers. Any product that may be evaluated in this article, or claim that may be made by its manufacturer, is not guaranteed or endorsed by the publisher.

## Footnotes

1
https://phenome.jax.org

2
https://CRAN.R-project.org/package=factoextra

## Abbreviations

GWAS: genome-wide association study
HBCGM: haplotype-based computational genetic mapping
Indel: insertion or deletion
PC: principal component
PCA: principal component analysis
PS: population structure
SNP: single nucleotide polymorphism.
